# Effect of income level on adherence to antidepressant treatment in first onset depression outpatients

**DOI:** 10.1371/journal.pone.0238623

**Published:** 2020-09-10

**Authors:** Nam-Ju Ji, Yeon-Pyo Hong

**Affiliations:** Department of Preventive Medicine, Chung-Ang University College of Medicine, Seoul, Republic of Korea; Chiba Daigaku, JAPAN

## Abstract

Clinical guidelines for depression management recommend continuous antidepressant medication; however, poor adherence to medication is commonly seen in the treatment of depression. Income is an important factor influencing antidepressant medication adherence. The purpose of this study was to investigate the relationship between income level and adherence to antidepressant medication in outpatients with depression. This was a retrospective cohort study using National Health Insurance claim data for services provided between January 1 and December 31, 2012. We examined data from a total of 142,336 individuals aged 18 years or older who were continuously enrolled in treatment after a new episode of major depression and who had initiated antidepressant treatment. The operational definition of adherence to antidepressant treatment was medication being dispensed to the patient at least 80% of the time during 3 and 6 months (ie. MPR ≥80%). To investigate the relationship between income level and adherence to antidepressants, we estimated adjusted odds ratios and 95% confidence intervals using hierarchical logistic regression analysis, adjusting for sociodemographic, clinical, and medical use characteristics. A total of 22.64% and 15.13% of depression patients showed adherence to antidepressants during 3 and 6 months, respectively. A statistically significant association was found between income level and adherence to antidepressants over 3 and 6 months for individuals with employee and self-employed subscribers. In addition, adherence to antidepressants was lower among those with a lower income than those with a higher income. We confirmed that social disparities exist in adherence to antidepressant treatment by income level in this Korean population-based retrospective cohort of depression outpatients.

## Introduction

Depression is a common disease with a lifetime prevalence of 13–16% among the world’s population and is considered a major public health problem in many countries [1–3]. Complying with an antidepressant treatment is considered very important for managing depression [4]. Clinical guidelines for depression management recommend continuous antidepressant medication, but early discontinuation of antidepressant treatment has been documented in various populations and clinical settings [5].

Existing research on correlates of adherence to antidepressants often focus on sociodemographic variables (gender, age, income status, fear of drug dependence, perception of treatment efficacy), clinical factors (severity, comorbidity), medical use behavior (characteristics of antidepressants, side effects, type of treatment), and provider characteristics (specialty, skill in diagnosing depression, prescribing appropriate antidepressant treatments, relationship with patient, amount of time spent in patient education). Income is an important factor in the continued use of antidepressants and in the selection of antidepressant type [6–10]. Previous studies on social disparities in terms of socioeconomic status (including income) and antidepressant treatment in depression patients found that early discontinuation was more frequent among patients of low socioeconomic status [7,9,11,12].

Although low socioeconomic status has been associated with increased duration of major depression, several studies have also shown the duration of antidepressant treatment to be shorter among individuals of low socioeconomic status [10–15]. In many studies, low income patients were more likely than higher income patients to be prescribed tricyclic antidepressants (TCA) [7,9,12], which may affect medication adherence and eventually lead to differences in treatment outcomes.

There is not enough research in the relationship between income status and antidepressant treatment. Even though Korea has the highest suicide rate in the world, research on adherence to antidepressants using representative data has not yet been conducted in this country. Therefore, this study was conducted to determine the association between income level and adherence to antidepressant medication in a Korean nationwide cohort of outpatients with depression.

## Materials and methods

### Study design and data source

This retrospective study analyzed data from the Korean National Health Insurance Database (NHID) from 2007 to 2014. The database includes data on patient demographics and clinical information such as disease diagnosis, drug prescriptions, and medical use information. Individuals were included in the study if they received their first diagnosis of depression in 2012. The index date was defined as the date diagnosis. The observation period for each patient was 2 years from the index date, and the end date of observation was December 31, 2014.

### Study population

The cases were defined as individuals who had their first diagnosis of depression (ICD-10 codes: F32.x, F33.x, F34.1) between January 1 and December 31, 2012, as outpatients, had not been diagnosed with depression in the previous 5 years, and had been prescribed more than one antidepressant.

Patients with related schizophrenia (F063, F20.x, F21.x, F232, F25.x) or mania (F30.x, F31.x, F34.0) were excluded from the study population. Medical aid patients were also excluded because, as their income was low, their treatment was free of charge. Therefore, these are exception in medical service use studies. In addition, patients who died during the study and patients whose main medical institutions were traditional medicine, dental, and pharmacy care were also excluded.

**Fig 1:**
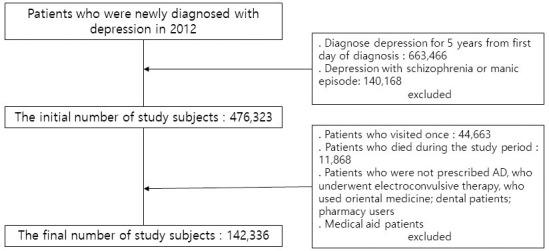
The process of selection of the research subjects.

A total of 1,181,173 outpatients were recorded in the NHID as being diagnosed with depression from January 1 to December 31, 2012. Of these, 663,466 patients who had been diagnosed with depression in the 5 years prior the index date were excluded, and 140,168 patients with schizophrenia or mania were also excluded. Therefore, the final number of study participants was 142,336. The process of selection of research participants is shown in Fig 1. The Institutional Review Board of Chung-Ang University approved the study protocol and consent form (IRB No. 1041078-201709-HRBM-185-01).

#### Income status

A square-root equivalence scale was used to compare the income class of the participants. For this purpose, the equalized personal income was calculated by dividing the household total income by the square root of household size [16].

The insurance fee calculation differs for employees and self-employed subscriber; thus, we analyzed these two groups separately. Insurance fee for employee subscribers are set based on their salary, while insurance fee for self-employed subscribers are set by combining their own assets, cars and property. The equalized personal income were classified into five class by the quintile. The highest income is Class 5 and the lowest income class is Class 1. It is assumed that there was no change in income level during the study period.

#### Covariates

Demographic, clinical, and medical use characteristics were selected as factors that may affect adherence to antidepressant medication. Demographic characteristics included gender, age at diagnosis of depression (onset age), area of residence, type of insurance (employee or self-employed subscriber), and type of employee subscriber (insured or dependent). The clinical characteristics included comorbid psychiatric illness, comorbid somatic illness, and depression severity at diagnosis. The medical use characteristics included initial antidepressants, initial department and medical institution, treatment type, number of antidepressant changes within 3 months from index date (adherence in 6 months), number of visits within 3 months from index date (adherence in 6 months).

In the case of comorbid psychiatric illness, we recorded personality and anxiety disorders, which are most relevant to depression. Depression severity is difficult to identify in health insurance claim data [17]; thus, we used the ICD-10 codes, and depression severity was defined as follows: F32.0 and F33.0 as mild; F32.1 and F33.1 as moderate; and F32.2, F32.3, F33.2, and F33.3 as severe. F32.8, F32.9, F33.4, F33.8, F33.9, and F34.1 were also considered mild.

Initial antidepressants were those prescribed at the time of diagnosis and were classified as TCA, selective serotonin reuptake inhibitor (SSRI), serotonin-norepinephrine reuptake inhibitor (SNRI), other, or combined (two or more antidepressants). Treatment type was divided into antidepressant-only treatment (single treatment) and drug treatment with psychotherapy (combination treatment). Combination treatment was the case when a combination of medication and psychotherapy was performed in the month of the claim, or claims for both types of treatment within the study period. The frequency of antidepressant changes and outpatient visits were examined based on 3 months of the acute phase.

#### Adherence to antidepressant

The medication possession ratio (MPR) was used as the index of adherence to antidepressant treatment; MPR was defined as the total number of days a medication was actually dispensed to patients divided by the number of treatment days. We considered a participant adherent to antidepressant treatment if medication was dispensed at least 80% of the time (MPR ≥80%) during the first 3 and 6 months following treatment initiation [18]. Once the antidepressant medication was dispensed, the patient was assumed to take it as prescribed.

### Statistical analyses

The chi-square test was used to compare the distribution of demographic, clinical, and medical use characteristics according to the income level of the participants (P <0.05).

We analyzed the association between income status and adherence to antidepressants during 3 and 6 months following first prescription using hierarchical logistic regression with adjustment for demographic, clinical, and medical use characteristics in order. The analysis was thus conducted using four models: Model 1 analyzed only the relationship between income class and adherence to antidepressants without adjusting for any other variables; Model 2 added the demographic characteristics to Model 1; Model 3 added the clinical characteristics to Model 2; and Model 4 added the medical use characteristics to Model 3. The analysis variables for each model are shown in Table 1. The fitness of the model was tested with Hosmer-Lemeshow’s X^2^ statistics, and the C-statistics for evaluating the predictive and explanatory power of the models were also calculated.

**Table 1:** Variables by model.

Model	Variable
Model 1	Income class
Model 2	Income class, demographic characteristics (gender, onset age, area, type of insurance, type of employee insurance)
Model 3	Income class, demographic characteristics (gender, onset age, area, type of insurance, type of employee insurance), clinical characteristics (comorbid mental illness, comorbid somatic illness, severity of depression)
Model 4	Income class, demographic characteristics (gender, onset age, area, type of insurance, type of employee insurance), clinical characteristics (comorbid mental illness, comorbid somatic illness, severity of depression), medical use characteristics (initial antidepressant, initial department, initial medical institution, treatment type, number of antidepressant changes within 3 months from index date, number of visits within 3 months from index date)

In a logistic regression, if the value of the dependent variable is over 10%, there is a risk that a difference between odds ratio (OR) and relative risk (RR) can occur. In this case, OR should be changed to RR [19]. In this study, the adherence to antidepressant medication during 3 months was 22.64%, and that during 6 months was 15.13%. Therefore, RR is presented in the results tables. Data analyses were carried out using SAS version 9.4.

## Results

### Sample characteristics

Of the final 142,336 participants, 91,148 were employee subscribers and 51,188 were self-employed subscribers. The general characteristics of the study participants are shown in Table 2. Class 1 (lowest income group) was the most frequent with 21.28% of participants, followed by Class 2 with 19.75%, Class 3 with 19.70%, Class 5 (highest income group) with 19.64%, and Class 4 with 19.62%. Except for Class 1, the percentage of individuals in the four income classes was about the same. In addition, differences in the number of participants were observed by characteristics (Table 2).

In the first year after the diagnosis of depression, only 4% of the patients changed their medical institutions, and thus most patients were treated at the initial medical institution.

**Table 2:** Characteristics of study population.

Variable		n	%
Income class	Class 1	30,292	21.28
	Class 2	28,118	19.75
	Class 3	28,047	19.70
	Class 4	27,930	19.62
	Class 5	27,949	19.64
Gender	Man	50,536	35.50
	Woman	91,800	64.50
Onset age	≤29	31,073	21.83
	30–49	49,354	34.67
	50–69	45,761	32.15
	≥70	16,148	11.34
Type of insurance	NHI Employee subscriber	91,148	64.04
	NHI Self-employed subscriber	51,188	35.96
Type of employee insurance	Insured	32,685	35.86
	Dependent	58,463	64.14
Area	Urban	130,562	91.73
	Rural	11,774	8.27
Comorbid mental illness	No	125,702	88.31
	Yes	16,513	11.60
Comorbid somatic illness	No	139,846	98.25
	Yes	2,490	1.75
Severity	Mild	93,048	65.43
	Moderate	38,381	26.99
	Severe	10,786	7.58
First antidepressant	TCA	15,901	11.17
	SSRI	75,314	52.91
	SNRI	5,604	3.94
	Other	23,441	16.47
	Combined	22,076	15.51
Treatment type	Single	119,666	84.07
	Combination	22,670	15.93
First department	Psychiatry	124,336	87.38
	Non-psychiatric	17,961	12.62
First health care institution	Clinic	101,735	72.25
	Hospital	39,080	27.75
Number of antidepressant changes within 3 months	0	24,452	17.18
	1	25,840	18.15
	≥2	92,044	64.67
Number of visits within 3 months	<3	75,210	52.84
	≥3	67,126	47.16
Change of medical institution within 1 year	No	136,260	95.73
	Yes	6,076	4.27
Total		142,336	100.00

*Class 1: lowest income group; Class 5: highest income group. TCA: tricyclic antidepressants, SSRI: selective serotonin reuptake inhibitor; SNRI: serotonin-norepinephrine reuptake inhibitor.

### Employee subscribers

The adherence to antidepressant medication during 3 and 6 months was 23.22% and 15.53%, respectively, among NHI employee subscribers. In this group, adherence to antidepressant medication during 3 and 6 months was significantly associated with income class in all models (Table 3). Adherence tended to decrease with decreasing income. In Model 4, the RR for adherence to antidepressants medication at 6 months was relatively lower than at 3 months, and the difference in RR of adherence between classes was smaller.

**Table 3:** Relationship between income level and adherence to antidepressants during 3 and 6 months for employee subscribers (N = 91,148).

Income class	Model 1^†^		Model 2^§^		Model 3^∥^		Model 4^¶^	
	RR	95% CI	RR	95% CI	RR	95% CI	RR	95% CI
**3 Months**								
Class 5	ref	-	ref	-	ref	-	ref	-
Class 4	0.95	0.92–0.99	0.95	0.91–0.98	0.95	0.91–0.98	0.95	0.91–0.99
Class 3	0.90	0.86–0.93	0.90	0.87–0.94	0.90	0.87–0.94	0.92	0.88–0.95
Class 2	0.86	0.83–0.89	0.88	0.84–0.91	0.88	0.84–0.92	0.88	0.85–0.92
Class 1	0.85	0.82–0.88	0.86	0.83–0.89	0.86	0.83–0.89	0.87	0.83–0.90
C-statistics	0.522		0.565		0.570		0.681	
**6 Months**								
Class 5	ref	-	ref	-	ref	-	ref	-
Class 4	0.92	0.87–0.96	0.92	0.87–0.96	0.92	0.87–0.96	0.89	0.85–0.94
Class 3	0.86	0.82–0.90	0.86	0.82–0.91	0.86	0.83–0.91	0.87	0.83–0.92
Class 2	0.84	0.79–0.88	0.86	0.82–0.91	0.86	0.82–0.91	0.87	0.82–0.92
Class 1	0.82	0.78–0.86	0.85	0.80–0.88	0.85	0.80–0.89	0.86	0.81–0.91
C-statistics	0.523		0.588		0.594		0.869	

Class 1: lowest income group; Class 5: highest income group; RR: relative risk; CI: confidence interval.

† Model 1,

§ Model 2,

∥ Model 3,

¶ Model 4

### Self-employed subscribers

The adherence to antidepressant medication at 3 and 6 months was 21.61% and 14.41%, respectively, in NHI self-employed subscribers. The relationship between income class and adherence to antidepressants in this group was different from that of employee subscribers. In Model 4, adherence during 3 months decreased from Class 5 to lower income classes and then increased again for Class 1 (Table 4). Adherence during 6 months tended to decrease from high-income to low-income classes in Model 4 only. However, adherence to antidepressants during 3 and 6 months was highest for Class 5, which was the highest income group, indicating that there was a significant association between income level and adherence to antidepressants.

**Table 4:** Relationship between income level and adherence to antidepressants during 3 and 6 months for self-employed subscribers (N = 51,188).

Income class	Model 1^†^		Model 2^§^		Model 3^∥^		Model 4^¶^	
	RR	95% CI	RR	95% CI	RR	95% CI	RR	95% CI
**3 Months**								
Class 5	ref	-	ref	-	ref	-	ref	-
Class 4	0.89	0.84–0.94	0.94	0.89–0.98	0.94	0.89–0.98	0.94	0.89–0.99
Class 3	0.84	0.80–0.88	0.90	0.86–0.95	0.90	0.85–0.95	0.89	0.84–0.94
Class 2	0.82	0.77–0.86	0.89	0.84–0.94	0.89	0.84–0.94	0.87	0.82–0.92
Class 1	0.90	0.85–0.95	0.95	0.91–1.01	0.95	0.91–1.01	0.91	0.86–0.96
C-statistics	0.524		0.569		0.576		0.695	
**6 Months**								
Class 5	ref	-	ref	-	ref	-	ref	-
Class 4	0.88	0.83–0.94	0.96	0.90–1.02	0.96	0.90–1.02	0.96	0.88–1.02
Class 3	0.82	0.76–0.88	0.91	0.85–0.97	0.91	0.84–0.97	0.91	0.84–0.98
Class 2	0.80	0.75–0.86	0.92	0.85–0.97	0.92	0.85–0.97	0.91	0.84–0.98
Class 1	0.87	0.82–0.93	0.96	0.90–1.02	0.96	0.90–1.02	0.90	0.83–0.97
C-statistics	0.524		0.590		0.597		0.878	

Class 1: lowest income group; Class 5: highest income group; RR: relative risk; CI: confidence interval.

† Model 1,

§ Model 2,

∥ Model 3,

¶ Model 4

## Discussion

In this study, the adherence to antidepressant medication during 3 months was 22.64%, and that during 6 months was 15.13% (MPR ≥80%). These results indicate that adherence to antidepressants is very low in patients with depression, which is consistent with previous studies. There was a significant association between adherence to antidepressants and income level for both employee and self-employed subscribers, except for some classes in self-employed subscribers. In addition, higher income patients were more likely than those with lower incomes to show adherence to antidepressant medication.

The difference in the RR of adherence to antidepressants between income classes was about 0.1, which is very small compared to the results of overseas studies. The RR of discontinuing antidepressants among low income individuals was 1.12–1.15 compared to high income individuals in a study using national insurance data in France [7]. The RR of adherence to antidepressants in the low income class was 0.64 compared to the high income class in a study using the panel survey data in US [11]. The RR of adherence of antidepressants among high income individuals was 1.23–1.25 compared with low income individuals in a study using private insurance data in US [3].

The difference in the RR of adherence to antidepressants at 3 months in employee subscribers was 0.05 between Class 5 & Class 4, and 0.13 between Class 5 & Class 1. That is, the difference with the lower layer is relatively greater than the difference between the upper layers. This means that the lower the income level, the higher the barriers to treating depression.

In general, SSRIs are known to show better medication compliance and fewer side effects than TCA [20]. Previous studies have reported that SSRIs tend to be prescribed in relatively higher income groups, while TCA is prescribed in lower income groups, and the results of the present study also indicate this [7,9,12]. In this regard, researchers suggested that this is related to drug prices, as SSRIs are relatively more expensive than TCA [7,12]. Such differences in drugs may lead to different side effects and treatment compliance, resulting in discontinuation of certain depression treatments and subsequent relapse.

The statistical significance did not change even if the effects of other variables were added step-wise to the analysis model. The model 4, including all variables, also showed a statistically significant relationship between income status and adherence to antidepressants. In the case of self-employed subscribers, no correlation was found in the order of income class. This may be due to the fact that the income level of self-employed subscribers is not accurately reflected, unlike that of employee subscribers, who are paid insurance fees based on their salary.

There are several limitations to this study. First, given the nature of insurance claim data, we could not measure other confounders as potential factors influencing adherence to antidepressant medication, such as level of education, psychological factors, life events, and past experiences and relationships with providers, as these were not collected.

Second, we did not check whether household members lived together when calculating income. For example, the correct number of cohabitants was not reflected for those who did not live with their parents. This raises concerns about whether participants’ income level was calculated correctly.

Third, we were unable to know whether patients actually took the dispensed antidepressant medication, or whether the discontinuation of the medication was due to recovery. Despite these limitations, the results of this study have a high external validity and generalizability because we used NHID cohort data covering the entire population of outpatients with depression in Korea. In addition, unlike clinic-based cohorts, our data included information about patients who moved or changed medical institutions.

In conclusion, this study confirmed that social disparities in adherence to antidepressant medication according to income level among outpatients with newly diagnosed depression. Effective management of depression is very important in national health policy, as untreated depression causes serious social problems such as suicide. This study is the first to study the relationship between income level and adherence to antidepressant medication in Korea.
